# The Choice of Leg During Pull Test in Parkinson's Disease: Not Mere Chance

**DOI:** 10.3389/fneur.2020.00302

**Published:** 2020-05-06

**Authors:** Francesca Spagnolo, Augusto Maria Rini, Pietro Guida, Sara Longobardi, Petronilla Battista, Bruno Passarella

**Affiliations:** ^1^Neurological Department, Antonio Perrino's Hospital, Brindisi, Italy; ^2^Scientific Clinical Institute Maugeri SPA SB, IRCCS, Institute of Cassano Murge, Bari, Italy

**Keywords:** Parkinson's disease, worse side, handedness, Pull Test, lateralization

## Abstract

**Background:** Parkinson's disease (PD) starts asymmetrically and it maintains a certain degree of asymmetry throughout its course. Once functional disability proceeds, people with PD can change their dominant hand due to the increased disease severity. This is particularly true for hand dominance, while no studies have been performed so far exploring the behavioral changes of lower limb utilization in PD according to the lateralized symptom dominance. In the current study, we aim to track the foot preference of participants with PD to respond to the Pull Test.

**Methods:** Forty-one subjects suffering from PD, with a H&Y scale ≤ 2, were recruited. A motor evaluation was performed, including the motor part of the MDS-UPDRS, its axial and lateralized scores (for more and less affected side), two Timed Tests, namely Time to Walk a standard distance (TW, in seconds) and Time Up and Go Test (TUG, in seconds), and the Pull Test. The preferred foot (right or left) involved in the step backward was recorded. Thirty-seven healthy controls underwent a motor assessment which included the Pull Test and the Timed Tests. Both participants with PD and controls were right-handed. To evaluate the relationship between the response to Pull-Test and PD-symptoms, subjects with PD were further divided into two groups: (1) Right more affected side (Right-MAS), and (2) Left more affected side (Left-MAS).

**Results:** Both groups of subjects with PD (Right-MAS and Left-MAS) during the Pull Test shifted significantly their leg use preference toward the opposite side than the more affected side: Right-MAS used preferentially their left leg (71%) and vice versa (*p* < 0.001). The limb preference shift was especially true for Left-MAS group that almost invariably used their right, dominant leg to respond to the Pull Test (95%). Similar results were obtained comparing people with PD and Controls.

**Conclusions:** This study shows that the limb used to respond to the Pull Test generally predicts the contralateral side of worse PD involvement. As the disease takes place, it prevails over hemispheric dominance: right-handed subjects with left side PD-onset and worse lateralization tend to be hyper-right-dominant, while right-handed subjects with right side PD-onset and worse impairment tend to behave as left-handers. Lateralization of symptoms in PD is still a mysterious phenomenon; more studies are needed to better understand this association and to optimize tailored rehabilitation programs for people with PD.

## Introduction

Parkinson's disease (PD) is an asymmetric disorder. Asymmetry is a key clinical feature especially at the beginning of the disease, even included in its diagnostic workout as a clinical parameter to differentiate PD from other parkinsonian syndromes (1). Moreover, a certain degree of asymmetric motor involvement is usually maintained throughout the PD course (2, 3) and the side of PD onset generally remains the worst affected one during disease progression. This clinical asymmetry corresponds to a neurochemical lateralization, and postmortem as well as *in vivo* studies show a correlation of motor involvement with contralateral dopaminergic deficit (4–6). The reason for PD asymmetry remains unclear (7). Several studies have addressed the link between dominant hand and the dominant side of PD symptoms, finding that the dominant side usually correspond to the side of PD-onset and worse motor involvement (8–11). Specifically, right-handed subjects suffering from PD showed a significant excess of right-dominant PD symptoms, while left-handed subjects with PD more often have left-dominant PD impairment (9, 11). The reason for the association between PD-symptoms and dominant hand side is unclear; however, we can speculate that an increased dopamine metabolism due to the augmented functional request of the dominant hemisphere can lead to increased oxidative damage, lysosomal dysfunction, and finally to neurodegeneration (12). Once the dopaminergic damage takes place, functional disability proceeds and people with PD can change their dominant hand in the presence of increased disease severity: pre-morbid right-handers with right side onset may change their preferred hand toward a left-hand preference instead. The same happens for pre-morbid left-handers with left side onset who may finally become right-handed (13).

To the best of our knowledge no studies have been performed so far exploring the behavioral changes of lower limb utilization in PD according to the lateralized symptom dominance.

In the current study, we aim to explore the foot preference of participants with PD to respond to the Pull Test. We hypothesized that, as for upper limbs, the side of PD onset may influence the preferred foot used to respond to this simple and meaningful clinical test.

The Pull Test is a quick and informative sub-score of the motor part of the Unified Parkinson's Disease Rating Scale (UPDRS), and it is used as the main clinical examination for evaluating postural stability in people with PD (14). It consists of pulling subjects backward from their shoulders and scoring the subject's ability to respond to this abrupt stimulus. External perturbations are present in everyday life and require adequate postural control to avoid falling. The score obtained at the Pull Test helps clinicians grading the severity of PD according to the Hoehn and Yahr (H&Y) staging scale (15).

Postural instability, abnormalities of posture, and gait disturbances can become a relevant component of parkinsonian motor disability, increasing the risk of falling. Among axial symptoms, postural instability is generally very difficult to treat (16) and sometimes only non-pharmacological therapies such as physical therapy and exercise may help to prevent falls (17). Indeed, the most reliable variable capable of estimating the risk of falls is the fall itself (18). However, many studies are ongoing to identify clinical batteries, kinematic assessments, or wearable sensors capable of identifying subjects with PD at risk of falling (19–21). Nevertheless, the measurements proposed are unlikely to be rapidly implemented into routine clinical practice. A more extensive understanding of what happens in people with PD before balance impairment takes place could be useful to develop targeted rehabilitative measure. In this study, we hypothesize that asymmetric motor involvement in PD could lead to different response to the Pull Test. We deliberately excluded subjects with postural instability, including only participants with a normal postural response, able to show a clear right or left response to the Pull Test.

We undertook this study to determine whether an association exists between lateralized symptom dominance and lateralization of the response to the Pull Test in a homogeneous population suffering from moderate PD and in healthy controls. We hypothesized that participants with PD with initial symptoms on their dominant side would use the contralateral leg to respond to the Pull Test and vice versa. Such information might be important to better characterize postural reflexes in a pre-falling phase and to implement more effective and targeted rehabilitation strategies in PD.

## Subjects and Methods

### Subjects

Forty-one consecutive right-handed subjects suffering from idiopathic PD according to United Kingdom PD Brain Bank criteria were recruited in our center from April to November 2019.

Inclusion criteria included: age < 80 years, H&Y scale ≤ 2, Mini-Mental State Examination (MMSE) ≥ 24/30, ability to provide oral and written informed consent.

Subjects were excluded if they had any psychiatric, neurological, orthopedic, or inflammatory disorder other than PD and if they underwent device-aided therapies for PD.

A control group of 37 right-handed healthy volunteers of similar age and sex, recruited mainly among PD subjects' spouses, were included as controls. Controls were excluded if they had any history of neurological disease, or any inflammatory or orthopedic-relevant problem. All participants with PD and controls reported right-hand dominance according to the Edinburg Handedness Inventory (22), a widely used tool to assess laterality. It is based on a questionnaire exploring the preferred side for both upper or lower limbs, as well as the preferred eye, in a series of daily life actions. Actions performed with the right (R) or the left (L) limbs are then summarized and the final score is obtained by this formula: (R – L)/(R + L). If the score is between 0.5 and 1, subjects are considered right-dominant. In this study we only included subjects who showed a right preference for both upper and lower limbs in the Edinburg Handedness Inventory.

Demographic and clinical data of the two groups are shown in Table 1. In people with PD the side of initial motor involvement was retrospectively recorded as well.

**Table 1:** Demographic and clinical characteristics of Controls and people with PD.

	**Controls**	**PD**	**Right-MAS**	**Left-MAS**	***p***	***p***
	***n*** **=** **37**	***n*** **=** **41**	***n*** **=** **21**	***n*** **=** **20**	***PD vs. Controls***	***Right-MAS vs. Left-MAS***
Age (years)	61 ± 11	65 ± 10	63.4 ± 8.9	65.8 ± 9.7	0.129	0.205
Male gender	57%	73%	71%	75%	0.128	0.802
Disease duration (years)		5.6 ± 4.6	4.8 ± 3.1	6.4 ± 5.7		0.608
LEDD (mg/day)		533 ± 453	490 ± 411	582 ± 507		0.945
H&Y (0–5)		1.97 ± 0.16	1.95 ± 0.21	2.0 ± 0.0		0.495
MDS-UPDRS III (0–132)		35 ± 13	33 ± 12	37 ± 13		0.286
Axial (0–36)		5.2 ± 3.0	4.9 ± 2.9	5.3 ± 3.2		0.688
RIGHT_side (0–36)		11.8 ± 4.8	13.6 ± 4.6	10.2 ± 4.6		**0.031**
LEFT_side (0–36)		13 ± 7	8 ± 5	16 ± 5		**<0.001**
Asymmetry index (0–100)		30 ± 22	34 ± 25	26 ± 18		0.464

*Data are shown as mean ± standard deviation, when appropriate, range values are shown. The bold value refers to statistically significant comparisons (p < 0.05). Right-MAS: participants with PD having worse motor scores on their right hemibody; Left-MAS: participants with PD with worse motor score on their left hemibody*.

This study was approved by our Ethics Commission and all subjects provided their written consent prior to enrolment.

### Clinical Evaluations

Clinical evaluation for participants with PD included the motor portion (part III) of the MDS Unified Parkinson's Disease Rating Scale (MDS-UPDRS) (23). MDS-UPDRS motor score was further divided according to PD lateralization in the more affected side (MAS) and the less affected side (LAS), obtained adding for each hemibody the items: 3.3–3.8 and 3.15–3.17 (range 0–36).

According to the side of worse involvement, subjects with PD were further divided in Right-MAS if MAS corresponded to the right, dominant hemibody, and Left-MAS if MAS corresponded to the left, non-dominant hemibody. A difference between MAS and LAS was also considered for each participant with PD, both in terms of absolute value (MAS-LAS) and as relative measurement (Asymmetry index), calculated with the following formula:

$$\begin{array}{l} \text{Asymmetryindex}=\left(\text{MAS}-\text{LAS}\right)/\left(\text{MAS}+\text{LAS}\right)\times 100. \end{array}$$

Axial involvement was independently considered adding the following items: 3.1–3.3a, 3.9–3.13, 3.17e (range 0–36).

As part of the MDS-UPDRS, Pull Test was performed three times during each evaluation. Subjects were instructed to stand in bipedal stance with the feet positioned at the pelvic width distance and eyes fixated at a target positioned in front of them. They were asked to respond to the Pull Test by performing a step backward, toward the examiner. Instructions were the same for each subject: “I am going to pull you backward. Try to keep your balance, you can step back if you need to.” The preferred foot (right or left) involved in the step backward was recorded for each subject during the three trial. A final side of response to the Pull Test was then obtained, considering the mode of the three trials.

To limit the variability, the Pull Test as well as the clinical assessments were conducted by the same examiner, with expertise in movement disorders, under the same experimental conditions. Agreement in the three Pull Test trials was also considered for every subject.

#### Timed Tests

Timed tests included Time to Walk Test(TW) and Time Up and Go Test (TUG) (24).

For people with PD, motor evaluation was carried out during their usual antiparkinsonian treatment (ON condition). Dosages of antiparkinsonian medications were recorded as well, and expressed as levodopa-equivalent daily dose (LEDD, mg) (25).

Controls underwent a motor assessment which included the Pull Test and the Timed Tests (TW and TUP) using the same experimental conditions as for participants with PD. Both subjects suffering from PD and controls were blind to the aim of the study.

### Statistical Analysis

Continuous variables were reported as mean ± standard deviation and compared by study groups with Student's *t*-test or Mann-Whitney *U*-statistic, when appropriate. Categorical data were described with absolute frequencies or percentage and the association was evaluated by using chi-square or Fisher's exact test. A *p* < 0.05 was considered statistically significant.

In order to evaluate the relationship between the response to Pull Test and PD symptoms, the 41 subjects with PD were further divided into two groups: (i) participants with PD worse on the right side (Right-MAS) and (ii) participants with PD worse on the left side (Left-MAS).

All analyses were conducted using STATA software, version 14 (Stata-Corp LP, College Station, Tex).

## Results

### Controls

In accordance with their right-handedness, controls mainly used their right leg to step backward during the Pull Test (68 vs. 32%). In the control group the agreement on the limb used during the three Pull Test trials was very high, 25/37 subjects (68%). Only 32% of controls did not use always the same leg to respond to the Pull Test (using once the contralateral leg). Specifically, in this group the inconsistent stepping response occurred: for two subjects (2/12; 17%) during the first trial, for four subjects (4/12; 33%) in the second trial, and for six participants (6/12; 50%) in the last trial.

### PD Group

In people with PD, the side of first motor signs appearance corresponded all the time with the side of worse PD involvement according to the UPDRS lateralized scores.

Subgroups of participants with PD based on dominant versus non-dominant side of motor symptoms, named Right-MAS and Left-MAS, did not significantly differ in their main clinical characteristics (age, disease duration, sex distribution, LEDD), as well as in the total motor UPDRS scores (33 ± 12 vs. 37 ± 13, for Right-MAS and Left-MAS, respectively). As expected, Right-MAS exhibited higher right lateralized motor scores than Left-MAS (RIGHT_side: 13.6 ± 4.6 vs. 10.2 ± 4.6; *p* = 0.031), while Left-MAS group showed worse motor involvement in their left hemibody, compared to Right-MAS (LEFT_side: 16 ± 5 vs. 8 ± 5; *p* < 0.001).

The asymmetry between MAS and LAS in people with PD subgroups was similar, both in terms of absolute value (MAS-LAS) than in relative measures (Asymmetry index). Axial scores did not differ as well in the two PD subjects' subgroups (Table 1).

It is clear that both groups of subjects with PD (Right-MAS and Left-MAS) during the Pull Test shifted significantly their leg use preference toward the opposite side than the MAS, i.e., right side onset participants (Right-MAS) used preferentially their left leg than their right leg (71 vs. 29%) and vice versa (*p* < 0.001). The limb preference adaptation was especially true for subjects with PD with left side onset (Left-MAS), who almost invariably used their right, dominant leg to respond to the Pull Test (95 vs. 5%, Table 2).

**Table 2:** Difference between controls and participants with PD.

	**Controls**	**Subjects with PD**	**More Affected Side (MAS)**		**PD vs. Controls**	**Right-MAS vs. Left-MAS**	**Right-MAS vs. Controls**	**Left-MAS vs. Controls**
			**Right-MAS**	**Left-MAS**				
	***n* = 37**	***n* = 41**	***n* = 21**	***n* = 20**	***p***	***p***	***p***	***P***
Age (years)	61 ± 11	65 ± 10	62 ± 9	67 ± 10	0.129	0.099	0.682	0.092
Male gender	57%	73%	71%	75%	0.128	0.796	0.268	0.173
Pull-Test					0.544	**<0.001**	**0.004**	**0.022**
Left	32%	39%	71%	5%				
Right	68%	61%	29%	95%				
TW (s)	17.0 ± 3.4	19.3 ± 5.0	19 ± 4	20 ± 6	0.097	0.483	0.167	0.115
TUG (s)	9.3 ± 2.5	9.5 ± 2.9	8.6 ± 2.0	10.5 ± 3.4	0.791	0.102	0.391	0.194

*PD-subjects were subdivided in two groups according to the more affected side (MAS): Right-MAS and Left-MAS*.

*Data are shown as mean ± standard deviation. The bold value refers to statistically significant comparisons (p < 0.05). Right-MAS, participants with PD having worse motor scores on their right hemibody; Left-MAS, participants with PD with worse motor score on their left hemibody; TW, time to walk a standard distance, in seconds; TUG, Time Up and Go Test, in seconds*.

Timed tests (TW and TUP, in seconds) did not significantly differ between Right-MAS and Left-MAS; this is not surprising as the two subgroups did not differ significantly in any other clinical or demographical characteristic.

As for Controls, for people with PD as well the three Pull Tests trials revealed a very high concordance: 30/41 participants with PD (73%) always used the same leg to step backward, while for only 11/41 subjects suffering from PD the leg used differed once. In this last group of PD-subjects, 4/11 (36.4%) showed the different leg response during the first trial, 1/11 (9.1%) in the second Pull Test, and 6/11 (54.5%) in the last trial.

No correlations were found between the foot preference and duration of disease, age, or other clinical-demographic characteristics in either of the subgroups.

### PD vs. Controls

People with PD and controls showed no significant differences in age or gender, despite a slightly younger age of controls. Comparing PD subjects (*n* = 41) and Controls (*n* = 37), no differences emerge in the use of a particular side during the Pull Test (right leg: 61% of PD-participants and 68% of controls; left leg: 39% of PD subjects and 32% of controls). However, examining the PD participants subgroups, the difference with controls emerges: Right-MAS mainly used their non-dominant leg during the Pull Test compared to controls (*p* = 0.004); while, as we already said, Left-MAS used almost all the time their right, dominant, leg (*p* = 0.022 vs. controls).

People with PD and controls did not differ in TUG and TW performance, although there is a trend for a better performance in TW in the control group. This is understandable considering both the moderate phase of the disease and the pharmacological ON state of subjects with PD. The comparable motor performances between participants with PD and controls further indicates that the two groups are well matched and that the difference we see in the Pull Test behavior is probably mainly due to PD motor lateralization.

## Discussion

Despite the advances in diagnostic and monitoring instruments (26), PD remains a clinical diagnosis, and a careful observation of people with PD is probably more informative than any other instrumental measure. As a common finding that may accompany other signs of parkinsonism, evaluation of the Pull Test response should be a part of any routine neurological examination, to detect early postural instability and PD-progression. From our results, it seems even more informative, able to rapidly disclose the side of worst PD-lateralization.

In our simple clinical study, we examined the limb used during the Pull Test in a PD population and in a group of age- and sex-matched controls. We further analyzed the different behavior according to the PD symptoms lateralization, finding that the limb used to respond to the Pull Test generally predicts the contralateral side of worse PD involvement.

Motor asymmetry is a common finding in PD, especially in its early and moderate stages, while it may become less prominent as the disease progresses (27). A link between dopaminergic asymmetry and hand preference has been suggested (28) and subjects suffering from PD seem to significantly change the premorbid right-hand preference toward using the left when the side of PD onset is on the right, and vice versa. Changes in hand preference may not necessarily be the simple consequence of behavioral convenience, but can be considered mediated by impaired dopamine pathways associated with PD.

In this study, the choice of the leg used to step backward during the Pull Test is not random and it seems influenced by the side of worse PD motor involvement. The present results support and extend our understanding of the utilization of right vs. left side in people with PD to respond to an external perturbation, as the Pull Test. As the disease takes place, it prevails over hemispheric dominance: right-side dominant subjects with left-side PD onset and worst lateralization tend to be hyper-right-dominant, while right-side dominant subjects with right-side PD onset and worst impairment, tend to behave as left-side dominant.

As hand preference could imply an increased metabolic demand with possible negative consequences of oxidative stress in the contralateral hemisphere, we can speculate that the change in side-dominance in PD-subjects can produce a kind of leveling effect, rebalancing hemispheric asymmetries, finally leading to a more symmetric PD.

To limit confounding factors and to make our results as general as possible, only right-handed subjects were included in our study. Further, studies could address the behavior of left-handed subjects with PD and controls in these simple experimental conditions. Right-side dominance was carefully assessed using a scale (22) which included items exploring not only upper limb, but also eyes and lower limb dominance.

Additionally, we decided to limit recruitment of PD subjects, including only participants in a mild to moderate disease stage. This choice is also due to a practical reason: H&Y > 2 imply an impairment of balance, making it hard to detect the leg chosen to inefficaciously respond to the Pull Test. Moreover, aim of our study was to understand the Pull Test response when balance is normal (before the beginning of falls). The relevance in understanding the mechanisms influencing the simple choice of which leg to use to maintain balance relies on the fact that the Pull Test is an immediate and universal clinical technique, carried out daily in clinical practice. The meaning of the different behavior between people with PD with worse right or left involvement is so far unknown; longitudinal studies could address it in order to understand its relevance for example in the development of falls, and to address targeted rehabilitative measures.

We found that subjects with left-side PD-lateralization almost invariably use the dominant right leg during the Pull Test. This is particularly interesting considering that left-side onset is associated with long disease and ambulatory PD survival (29). We can hypothesize that a clear asymmetry and a clear side-dominance in PD actually helps maintain balance and prevent falls. Our results may then provide some valuable suggestions for a tailored physical therapy in parkinsonian subjects in order to make parkinsonian hemibodies more asymmetrical. However, more studied are needed to better clarify the link between handedness, change of handedness, and PD.

In our study, Time Walking and TUG tests were performed to introduce a quantitative measure of motor performance to compare participants with PD and controls. The lack of a significant difference of TW and TUG in the two groups indicates that motor performances are quite similar; this is probably explained by the mild disease stage and the therapeutic ON state of PD-subjects. Comparable motor results between the two groups (people suffering from PD and controls) imply that the difference in what we observe during the Pull Test is probably mainly due to the lateralized PD-involvement. We found no correlations between the response to the Pull Test and clinical-demographic characteristics. This is not surprising, as the duration of the disease and other clinical-demographic parameters did not predict the amount of hand preference shift in previous studies as well (13).

Lastly, in our study, we tried to understand the behavior of subjects who did not show a 100% concordance in the three Pull Test trials. Although numerically very few, both participants with PD and controls showed that the discordant trial was the third in 50% of cases. This result can be explained by fatigue or by a sort of learning effect that made the subject more confident in the use of the contralateral limb to efficaciously respond to the Pull Test. Upcoming studies could benefit from this information by focusing only on the first or second response to the Pull Test, as the third repetition would seem to be the least informative one. In this regard, in the MDS-UPDRS, instructions given to perform the Pull Test with a first milder, not rated trial, could cause some important information to be lost.

Several limitations are evident in our study. First of all, the subjective nature of the Pull Test (30). Low resolution and non-linear outcomes are also characteristic of this test. Munhoz et al. (30) reported that 77.3% of the subjects with PD in a clinical trial (*n* = 66) were pulled too lightly from their shoulders, while in 36.4% of the cases the examiners did not allowed enough space for the PD subjects to recover from the pulling event. However, to limit the variability in our study, the Pull Test was always conducted by the same examiner under the same experimental conditions. Second, in our study we preferred to not include participants with orthopedic or inflammatory conditions, as they could at least partially influence the postural response. However, co-existent arthritic changes cannot be excluded solely by a clinical examination, and no X-ray evaluations were performed.

Third, participants with PD in the present study were in the early and moderate stages of PD, which made it impossible to generalize the results. However, as we explained, we limited the recruitment to H&Y ≤ 2 to be sure to have a clear postural response, as also supported by the very high level of agreement in the leg used to step backward both in subjects with PD and in controls. Our results further support the utility of the Pull Test as a simple clinical method in everyday practice. According to our results, it gives important information not only about balance, but also about PD-lateralization. Future attempts to short MDS-UPDRS should consider including the Pull Test as a highly informative clinical instrument.

In conclusion, this study shows that PD influences the limb preference during external perturbations, such as the Pull Test. The direction of this influence is contralateral to the side of the body where the first signs of PD occur. Lateralization of symptoms in PD is still a mysterious phenomenon, surely associated with dominance; moreover, it provides several hypotheses concerning PD onset itself. However, more studies are needed to better understand this association and to optimize tailored rehabilitation programs for people with PD.

## Data Availability Statement

The datasets generated for this study are available on request to the corresponding author.

## Ethics Statement

The studies involving human participants were reviewed and approved by A. Perrino's Ethical Committee. The patients/participants provided their written informed consent to participate in this study.

## Author Contributions

FS: data acquisition and analysis, interpretation of results, and manuscript preparation. AR: study concept and design, interpretation of data, and manuscript revision. PG: statistical analysis and critical revision. SL: data acquisition and analysis. PB and BP: critical revision of manuscript for intellectual content.

## Conflict of Interest

The authors declare that the research was conducted in the absence of any commercial or financial relationships that could be construed as a potential conflict of interest. The reviewer, MD, and handling editor declared their shared affiliation at the time of the review.
